# Relationship satisfaction in the early stages of the COVID-19 pandemic: A cross-national examination of situational, dispositional, and relationship factors

**DOI:** 10.1371/journal.pone.0264511

**Published:** 2022-03-03

**Authors:** Julia Vigl, Hannah Strauss, Francesca Talamini, Marcel Zentner

**Affiliations:** Department of Psychology, University of Innsbruck, Innsbruck, Austria; University of Connecticut, UNITED STATES

## Abstract

The outbreak of the COVID-19 pandemic has had a large impact on various aspects of life, but questions about its effects on close relationships remain largely unanswered. In the present study, we examined perceived changes in relationship satisfaction at the beginning of the COVID-19 pandemic by using an international sample of 3,243 individuals from 67 different countries, mostly from Italy, the United States, the United Kingdom, Germany, Austria, and Switzerland. In April and May 2020, participants responded to an online survey that included questions about relationship satisfaction, their satisfaction before the pandemic, other relationship aspects (e.g., shared time), special circumstances (e.g., mobility restrictions), and enduring dispositions (e.g., insecure attachment). A decline in time shared with one’s partner was most strongly associated with perceived decreases in relationship satisfaction, resulting in a different pattern of findings for cohabiting and non-cohabiting individuals. Among the most influential moderators were anxious and avoidant attachment. The findings offer insights into both aggravating and protecting factors in couples’ responses to pandemic-related stressors.

## Introduction

Unlike any other event in recent history, the COVID-19 pandemic has had an impact on couples worldwide through major changes in everyday routines, including increasing levels of remote work, changes in childcare requirements due to closed childcare facilities and schools [1], and increased financial vulnerabilities due to job cuts [2]. To our knowledge, no studies have examined the effects of previous pandemics (e.g., the 2009/2010 H1N1 pandemic) on relationship satisfaction. Although there have been conceptual contributions and preliminary data from the United States [3, 4] Germany [5], and Romania [6] on the current COVID-19 pandemic, this study is the first to examine changes in relationship satisfaction in a large-scale, cross-national sample of cohabiting and non-cohabiting individuals. In addition, although previous COVID-19 studies have focused on specific factors, such as attachment style [7], we took a more comprehensive approach, ranging from individual dispositions to pandemic-related external stressors. Including both types of couples (cohabiting vs. non-cohabiting) in a cross-national comparison is important for understanding the generalizability of findings, as well as for providing insight into potential implications of the different self-isolation measures implemented across countries.

Although these measures are likely to have affected close relationships through known psychological mechanisms, not much is known about the role of certain types of external stressors (e.g., enforced closeness, travel bans) that are likely to have played an outsized role during the pandemic. In examining how couples’ relationship satisfaction has been affected by the pandemic, we therefore paid particular attention to pandemic-specific challenges, while drawing from previous theory and research about the effects of external stress on close relationships.

### The COVID-19 pandemic as a source of stress in close relationships

High levels of external stress are known to be associated with a decrease in relationship satisfaction, as they might spill over into the relationship and cause internal stress (see Randall and Bodenmann [8] for a review). In addition to the aforementioned disruptions to everyday routines, uncertainty about the pandemic’s length and impact, fear for one’s own health and that of loved ones [9], and emotional exhaustion [10] posed additional challenges. These external stressors could have led to problem behaviors such as rigidity, fear, or hostility in one or both partners, thereby compromising the quality of their interactions and eroding relationship satisfaction [11].

For cohabiting couples, one of the most salient consequences of the pandemic was a sudden increase in shared time resulting from measures such as curfews or remote work. The relational effects of this enforced closeness are difficult to gauge from previous research. Although some studies suggest that an excess of shared time and activities can lead to more stress and boredom in the relationship, especially when no specific activities are undertaken [12], others suggest that time spent together is associated with less intradyadic stress [13, 14]. Whether plenty of shared time is experienced as a benefit or a burden may depend on a variety of factors, such as couples’ intradyadic stress levels, resourcefulness in using shared time, and skills in coping with external stressors [11].

In contrast to the increase in shared time experienced by cohabiting couples, non-cohabiting couples likely experienced a decrease in time spent together because of mobility restrictions and travel bans [15]. Being deprived from seeing one’s romantic partner could lead to relationship dissatisfaction, as greater distance, greater time between visits, and the feeling of loneliness are linked to poorer relationship outcomes [16, 17]. On top of this, another factor of interest is sexual satisfaction because whereas the lack of psychological proximity with one’s partner can partly be compensated by means of video and other communication tools, such compensation is more difficult to achieve with regard to physical intimacy. Because dissatisfaction with the frequency of sex has been shown to have a negative impact on sexual and relationship satisfaction [18], the relationship satisfaction of non-cohabiting couples may have been more adversely affected than that of cohabiting couples.

### Moderators of stress in close relationships

According to the vulnerability-stress-adaption model [19], the experience of stressors can be both mitigated and aggravated by enduring vulnerabilities, leading to more or less adaptive processes that, in turn, affect relationship satisfaction and duration over time [19]. A large body of theory and research suggests that insecure attachment and negative emotionality play an important role in partners’ reactions to stress. For example, in contrast to securely attached individuals, people with anxious attachment have been found to use ineffectual coping strategies, such as self-reproach or negative outbursts of emotion, or to report more relationship conflicts [20]. Avoidant individuals, in turn, show a tendency to distance themselves and refrain from seeking support when problems arise, which has also been found to be associated with lower relationship satisfaction (for a review, see Shaver and Mikulincer [21]). Similarly, negative emotionality has been found to predispose partners to experience more conflict in close relationships, or to interpret innocent behaviors as hostile, which can be partly attributed to the use of ineffectual coping strategies characterized by either emotion-focused processing or disengagement [19, 22].

Paradoxically, stress does not always have a negative impact on relationship satisfaction. Attachment theory suggests that, in the face of an existential threat, couples tend to seek (physical) closeness and security in their partner and avoid conflict, resulting in a stronger and intensified relationship. This process allows couples to buffer fear through mutual protection and emotional comfort [23, 24]. In the case of the pandemic, the individual experience of threat could therefore have promoted proximity seeking and relationship consolidation.

### The current study

In summary, the focus of the current study was to investigate (a) whether satisfaction in close relationships changed at the beginning of the COVID 19 pandemic compared with retrospectively assessed relationship satisfaction before the pandemic, (b) which factors were involved in the perceived changes, and (c) how changes and predictors differ between cohabiting and non-cohabiting individuals. To this end, we conducted a cross-national study, in which participants responded to an online survey that included questions designed to cover the multiple challenges outlined above. Relationship satisfaction before the pandemic was assessed retrospectively in this study due to the lack of real-time data before the event. The use of retrospective assessments is a relatively common approach to the examination of the effects of unforeseen events (e.g. terror attacks; other studies on the impact of the COVID-19 pandemic [25–27]). Although retrospective data have their limitations, they have shown to be generally quite accurate in the case of relationship satisfaction [28].

In order to represent differences concerning severity of mobility restrictions, and to obtain results that can be generalized beyond a particular national context, we targeted participants in the United States, the United Kingdom, Italy, and the GSA region (Germany, Switzerland, and Austria). The GSA countries were combined into one group because of their cultural, historical, linguistic, and economic bonds, as well as the similarities in pandemic-related aspects, such as infection rates and restriction policies [29].

Based on previous research, we expected the following:

1. Relationship satisfaction would decrease at the beginning of the pandemic compared with the retrospectively assessed relationship satisfaction before the pandemic because of multiple external stressors triggered by the pandemic.
2. The abrupt, substantial changes in shared time between partners related to mobility restrictions would be related to a perceived decrease in relationship satisfaction, in particular in sexual satisfaction, for non-cohabiting partners because of limited contact opportunities; we were agnostic as to how the enforced increase in shared time would affect cohabiting couples.
3. Based on the vulnerability-stress-adaptation model, we predicted perceived change in relationship satisfaction to be affected by previously established risk factors (e.g., negative emotionality, anxious and avoidant attachment), as well as by pandemic-related factors (e.g., concerns about the pandemic, strength of mobility restrictions). In addition, we expected anxious and avoidant attachment and high negative emotionality to exacerbate the effects of pandemic-related stressors.

## Method

### Participants

A total of 3,557 people completed at least 80% of the survey, which includes all relevant questions for this study. Some participants were excluded, as they reported not being in a relationship at the time of assessment (*n* = 274) or not answering honestly and attentively (*n* = 21). In addition, participants indicating non-binary as their gender (*n* = 19; 0.6%) were excluded from the current analyses, as group comparisons are not possible with such a small sample, and gender was also included in the regression model. The final sample thus consisted of 3,243 individuals (73.4% women). The average age was 31 years (*SD* = 11.21, range = 18–79). About half of the participants lived with their partners (56.3%), 28.7% lived apart but in the same region, and 15.0% lived in different regions. Twenty percent reported having children. The average duration of the relationship was 6.30 years (*SD* = 8.00, range = 0.1–56.6). Seventy-three percent were in a committed relationship, 23.4% were married, and 3.7% were in an open relationship. Participants from 67 different countries responded to the survey, with the vast majority living in Italy (*n* = 1,094), the United States (*n* = 776), the United Kingdom (*n =* 302), and the GSA region, namely, Austria, Switzerland, and Germany (*n* = 775).

Possible biases in our samples were examined by comparing levels of age, gender, education, cohabitation, sexual orientation, and relationship satisfaction against those of the respective general populations. Our samples did not differ substantially from the general population values in regard to sexual orientation and relationship satisfaction (see S1 Table in S1 File for the respective comparisons).

### Measures

The online survey was provided in English, Italian, and German. When questionnaires were not available in Italian or German, they were translated by three independent translators who were fluent in the relevant languages.

#### Relationship and sexual satisfaction

The Relationship Assessment Scale [30] (German version by Hassebrauck [31]), a seven-item questionnaire for all types of romantic relationships, was used to measure relationship satisfaction. A one-item sexual satisfaction measure was added to it (“How sexually satisfied have you been/are you with your partner?”) [32]. Participants were asked to provide two separate ratings: one retrospective rating of their relationship satisfaction before the outbreak of the pandemic, and one rating of their current relationship satisfaction. The answers were given on a 5-point scale, with higher scores indicating higher relationship satisfaction. Internal consistencies of the eight-item measure were α = .88, .89, and .91 for the German, Italian, and English versions, respectively. Henceforth, we report only the minimum and maximum alpha values (computed from the different language versions of our survey).

#### Insecure attachment

The Experiences in Close Relationships questionnaire [33] was used to measure insecure attachment. From the recommendations for shortening this questionnaire by Lafontaine et al. [34], anxious attachment was assessed by the following four items: “I worry about being abandoned”; “I worry a fair amount about losing my partner”; “I need a lot of reassurance that I am loved by my partner”; “I worry that romantic partners won’t care about me as much as I care about them” (α = .78 to .89). Avoidant attachment was measured by the following four items: “I don’t feel comfortable opening up to romantic partners”; “I tell my partner just about everything”; “I feel comfortable sharing my private thoughts and feelings with my partner”; “I feel comfortable depending on romantic partners” (α = .63 to .77).

#### Personality traits

We used the extra-short form of the Big Five Inventory, which consists of 15 items [35] (German version by Rammstedt et al. [36]). Cronbach’s alphas were similar to or even above the values reported by Rammstedt et al. [36] for all five dimensions: Negative Emotionality (α = .71 to .74), Extraversion (α = .47 to .51), Agreeableness (α = .50 to .53), Conscientiousness (α = .55 to .58), and Openness (α = .48 to .50).

### Pandemic-related measures

#### Restrictions and management

We asked about the degree of mobility restrictions (1 = *no restrictions*, 4 = *strong restrictions*) and the duration of these restrictions (in days). In addition, we assessed various pandemic-related worries (see S3d Table in S1 File) on a 10-point scale and combined them into a single estimate of overall pandemic-related worries (1 = *not worried at all*, 10 = *very worried*; α = .70 to .74). We also collected data on approximately how many hours per week the participants spend outside their homes.

#### Change in shared time

Changes in frequency of interactions and joint activities with the partner relative to the time before the pandemic were assessed by asking about changes in frequency of physical contact (-3 = *less than half of the time*, 0 = *same as before the pandemic*, 3 = *twice as much*) and in frequency of conversations, joint activities, sex, and arguing, all on a scale that ranged from -4 (*much less than before the pandemic*) to 0 (*same as before the pandemic*) to 4 (*much more than before pandemic*). A principal component analysis suggested two factors that explained 74.5% of the variance: The first component comprised physical contact, joint activities, conversations, and sex, whereas the second component comprised arguments only. Because the analyses use directional change scores (see Data Analysis subsection), change variables are defined in terms of reported increase or decrease. In light of a general perceived decrease in shared time (see Changes in Shared Time and Shared Activities subsection), the four variables (change in frequency of physical contact, of joint activities, of conversations, and of sex) were reversed (higher values indicate less contact than before the pandemic; lower values indicate more contact than before the pandemic) and combined into a composite index termed decrease in shared time (α = .79 to .83). The item increase in arguing was left unchanged (higher levels indicate perceived increase in arguing). A final question assessed how much time the participants had for themselves before and during the pandemic from -4 (*much less than before*) to 0 (*same as before*) to 4 (*much more than before*). This variable was called increase in time for oneself.

#### Housing and professional situation

In addition to gender, age, country of residence, and nationality, the survey also asked about the size of the living area (rural, midsize rural, large urban), the home size (in square meters), access to private outdoor spaces (0 = *no*, 1 = *yes*), and satisfaction with degree of privacy available at home (1 = *very dissatisfied*, 5 = *very satisfied*). We also asked respondents to indicate whether they and their partner were employed before and during the pandemic (0 = *no*, 1 = *yes*) and whether the crisis had changed their job situation (e.g., decreased working hours, loss of job for self or for partner). Other measures were administered in the context of data collection, which are not relevant for the analyses reported here.

### Procedure

The study was conducted as an online survey by using the software *LimeSurvey*. Participants were recruited via the project website and through different media channels, including local newspapers, radio programs, social networks, podcasts, newsletters, and the SurveyCircle website, which allows sharing of research projects and mutual participation in questionnaires. In the United States and the United Kingdom, these methods of recruitment were supplemented with Clickworker, a paid crowdsourcing service, to attain a sufficiently large sample size. The participants recruited via crowdsourcing represented 12% (*n* = 394) of the entire sample and were compensated with 2.30 USD. Although these participants differed from the rest of the (respective) national sample in some aspects, their standing on key variables, such as relationship satisfaction and amount of contact change (see S2 Table in S1 File) did not. The survey was published online on April 8, 2020, and remained accessible for two months. Ninety percent of the participants took part in the study up to May 1, 2020 (*n* = 2,890, median = April 22, 2020), 99% up to May 15, 2020 (*n* = 3206), and the last 34 participants between May 16, 2020, and June 9, 2020. Participation was open to all individuals (minimum age 18 years old) who were currently in a relationship and gave their written informed consent to participate. The study was approved by the Ethics Committee of the Institute of Psychology, University of Innsbruck.

### Data analysis

To address whether there was any perceived change at all in relationship satisfaction among cohabiting and non-cohabiting individuals (hypotheses 1 and 2), we used a mixed repeated measures analysis of variance (ANOVA). A significant interaction between the categorical predictor (between subjects) and relationship satisfaction over time (within subjects) was interpreted to indicate an effect of the categorical predictor on the *mean level of changes* in relationship satisfaction over time. Multivariate regressions were used to identify the *predictors* of perceived change in relationship satisfaction. To assess the associations of predictors with retrospectively reported changes in relationship satisfaction in these analyses, we regressed residualized change scores for relationship satisfaction before and after the outbreak of the pandemic on predictors described under the Measures subsection. Residualized change scores provide a directional variable of change and their advantages include a reduction of selection and unobserved variable bias [37], as well as an attenuation of regression to the mean [38]. Positive scores indicate a perceived increase in relationship satisfaction, whereas negative scores denote a perceived decrease. Thus, predictors of change in relationship satisfaction with a positive beta-weight can be interpreted as having contributed to a perceived *improvement* in relationship satisfaction in the first weeks after the outbreak of the pandemic, whereas predictor variables with a negative weight can be interpreted as having contributed to a perceived *decline* in relationship satisfaction. A post hoc power analysis performed with the software g*power [39] showed an achieved power (1-β err prob) of .99

## Results

### General trends in relationship satisfaction: Hypotheses 1 and 2

#### Relationship satisfaction before and during the pandemic

**Fig 1:**
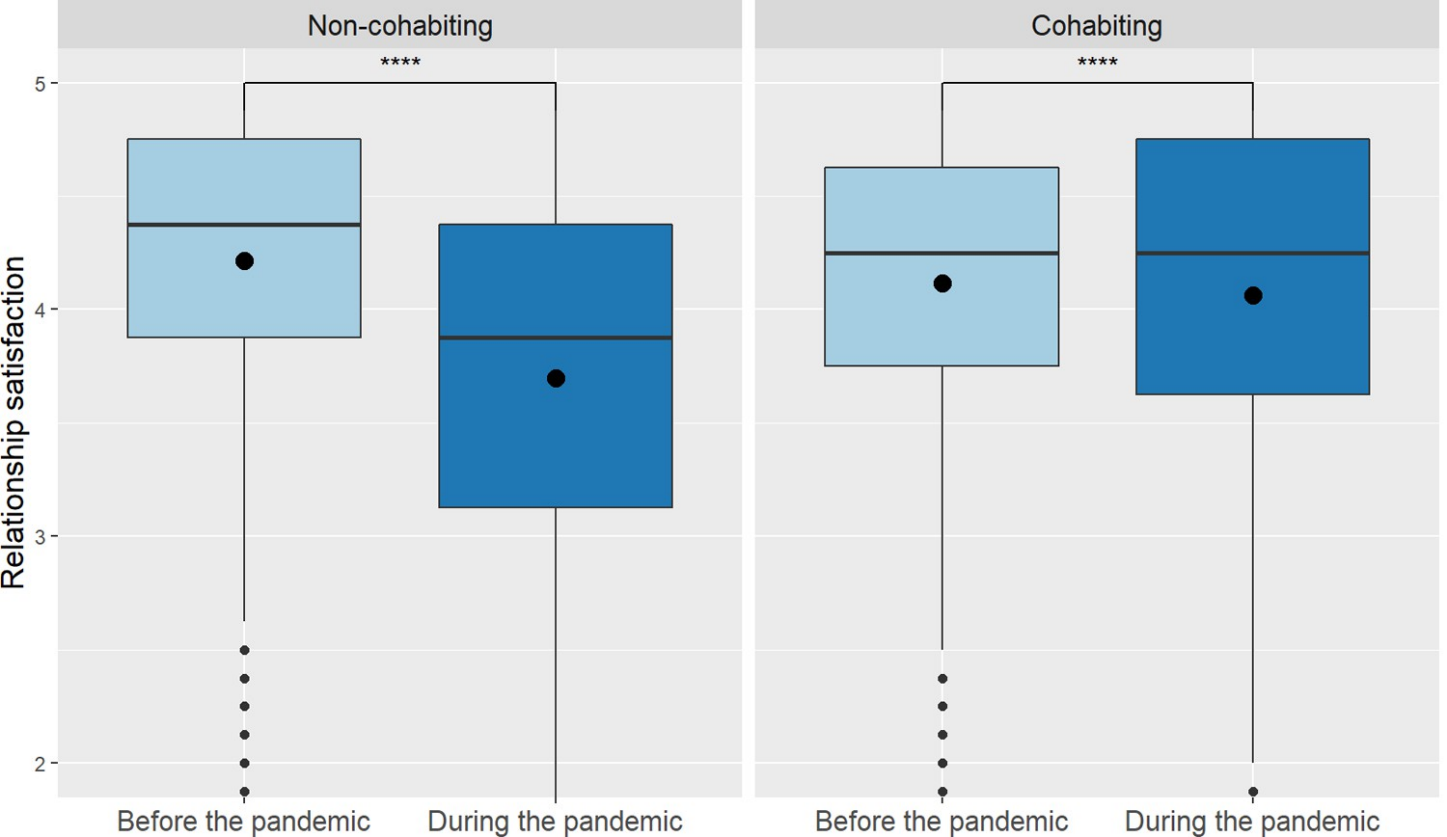
Relationship satisfaction before and during the pandemic according to cohabitation of participants. Asterisks show significant effects (****p < .0001); circles indicate arithmetic means.

Using a 2 (time: before and during the pandemic) × 2 (cohabiting status: cohabiting, non-cohabiting) mixed repeated measures ANOVA, we found a main effect of time, *F*(1, 3241) = 689.44, *p <* .001, η^2^ = 0.18, indicating that, overall, relationship satisfaction decreased at the beginning of the pandemic relative to individuals’ reminiscence of their relationship satisfaction before the pandemic. We also found a small main effect of cohabiting status, *F*(1, 3241) = 26.91, *p* < .001, η^2^ = 0.01, and an interaction between cohabiting status and time, *F*(1, 3241) = 465.15, *p <* .001, η^2^ = 0.13, implying that relationship satisfaction decreased more strongly for non-cohabiting individuals than it did for cohabiting ones (see Fig 1).

**Fig 2:**
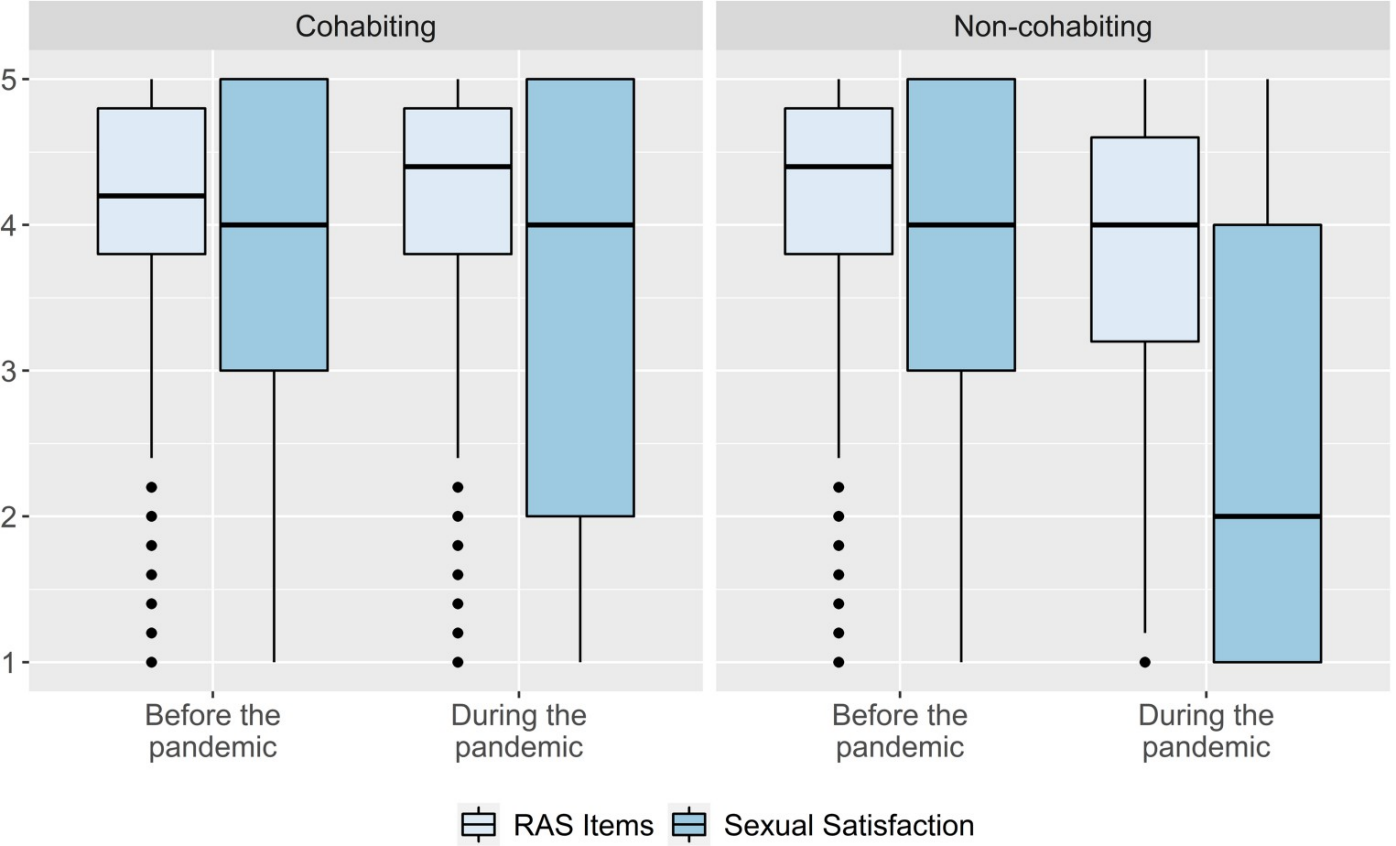
Changes in Relationship Assessment Scale (RAS) items and sexual satisfaction among cohabiting and non-cohabiting individuals. *n*_cohabiting_ = 1,825; *n*_non-cohabiting_ = 1,418.

In line with our assumption that sexual satisfaction might have suffered more than relationship satisfaction overall, especially in non-cohabiting couples, we compared the perceived decrease in general satisfaction (the seven items of the Relationship Assessment Scale [30]) with the perceived decrease in sexual satisfaction (one item [32]) across cohabiting and non-cohabiting couples. To this end, we ran a 2 (Relationship Assessment Scale time) × 2 (sexual satisfaction time) × 2 (cohabiting vs. non-cohabiting) repeated measures ANOVA. As indicated by a significant interaction between perceived relationship satisfaction change and sexual satisfaction change, sexual satisfaction did decrease more strongly than did relationship satisfaction overall, *F*(1, 3241) = 1070.96, *p <* .001, η^2^ = 0.25. Notably, and in line with our expectations, we found a three-way interaction between overall relationship satisfaction change, sexual satisfaction change, and cohabiting status, indicating that the stronger decline in sexual satisfaction relative to the general relationship was mainly experienced by non-cohabiting individuals, *F*(1, 3241) = 734.88, *p <* .001, η^2^ = 0.19 (see S4 Table in S1 File for details on the ANOVA and S5 Table in S1 File for an item analysis). Fig 2 provides an illustration of the three-way interaction.

#### Changes in shared time and shared activities

**Fig 3:**
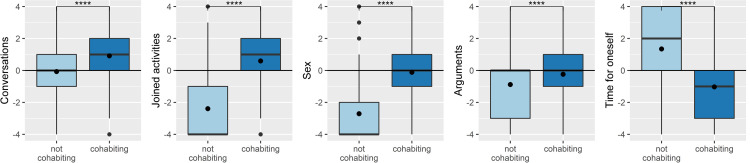
Changes in the frequency of different activities in non-cohabiting and cohabiting individuals (0 = no change, < 0 less frequent, > 0 more frequent). Asterisks show significant effects (****p < .0001); circles indicate arithmetic means.

An important reason for expecting a greater decline in relationship and sexual satisfaction in non-cohabiting compared with cohabiting couples was that mobility restrictions would result in a decrease of shared dyadic activities. As illustrated in Fig 3, this is indeed what we found. Whereas frequency of joint activities and sex decreased strongly among non-cohabiting individuals, cohabitants reported a slight increase in conversations and joint activities. As a mirror image of this pattern, time for oneself increased for non-cohabiting individuals but decreased for cohabiting individuals. Additional analyses on further pandemic-related factors (e.g., occupational changes, outdoor time, worries regarding the pandemic) can be found in S3a-S3e Table in S1 File.

### Predictors of perceived changes in relationship satisfaction: Hypothesis 3

To investigate the associations of the entirety of individual predictors and interactions among predictors with changes in relationship satisfaction, we first entered all predictors described in the Measures subsection in a multivariate regression. The complete parameter estimates are provided in Table 1, Model 1. Decreased shared time was the strongest predictor of perceived decline in relationship satisfaction, followed by increased arguing. The negative β coefficients indicate that a decrease in shared time and an increase in arguing predict a perceived decrease in relationship satisfaction. Other significant predictors were smaller in size and included pandemic-related worries, avoidant and anxious attachment, a decrease in working hours (negative impact), relationship duration, and the number of hours spent outside home (positive impact) (see Table 1). Although there was reason to believe that restriction level might have limited contact opportunities and thus acted as a source of external stress, it had no effect on perceived change in relationship satisfaction.

**Table 1:** Two models identifying factors related to perceived changes in relationship satisfaction after the outbreak of the pandemic.

	Model 1			Model 2		
Variable	*B*	*SE B*	β	*B*	*SE B*	β
Decrease in shared time^a^	-.73	.02	-.60**	-.73	.02	-.60**
Increase in arguing^a^	-.11	.01	-.23**	-.11	.01	-.23**
Pandemic-related worries	-.03	.01	-.06**	-.04	.01	-.06**
Avoidant attachment	-.04	.01	-.06**	-.04	.01	-.06**
Increase in time for oneself^a^	.02	.01	.06**	.02	.01	.06**
Feeling of privacy at home	.04	.01	.05**	.04	.01	.05**
Relationship duration	.01	< .01	.05*	.01	< .01	.05*
Anxious attachment	-.02	.01	-.04**	-.02	.01	-.04*
Decrease in working hours (self)^a^	-.08	.03	-.04**	-.09	.03	-.04**
Hours per week spent outside home	< .01	< .01	.03*	< .01	< .01	.03*
Living in the GSA region	.07	.04	.03^†^	.07	.04	.03
Living in the United States	.07	.05	.03	.07	.05	.03
Loss of employment (partner)^a^	-.09	.04	-.03*	-.09	.04	-.03*
Living in the United Kingdom	-.09	.06	-.03	-.09	.06	-.03
Decrease in working hours (partner)^a^	-.09	.06	-.02^†^	-.09	.06	-.02
Cohabiting	-.04	.04	-.02	-.04	.04	-.02
Living in other countries	.07	.06	.02	.06	.06	.02
Agreeableness	-.02	.02	-.02	-.03	.02	-.02
Loss of employment (self)^a^	-.05	.04	-.02	-.06	.04	-.02
Size of the living area	-.02	.02	-.02	-.02	.02	-.02
Home size	< .01	< .01	-.02	< .01	< .01	-.02
Education	-.01	.01	-.01	-.01	.01	-.01
Restriction level of the country	.03	.03	.01	.03	.03	.01
Extraversion	-.01	.02	-.01	-.01	.02	-.01
Gender	-.02	.03	-.01	-.01	.03	< .01
Living with children	.02	.04	.01	.02	.04	.01
Duration of pandemic-related restrictions	< .01	< .01	-.01	< .01	< .01	-.01
Conscientiousness	-.01	.02	-.01	-.01	.02	-.01
Access to a private outdoor space	.01	.04	< .01	.01	.04	< .01
Openness	< .01	.02	< .01	-.01	.02	-.01
Negative emotionality	< .01	.02	< .01	< .01	.02	< .01
Age	< .01	< .01	< .01	< .01	< .01	< .01
Decrease in shared time × anxious attachment				.06	.02	.05**
Decrease in shared time × avoidant attachment				.05	.02	.04**
Decrease in shared time × negative emotionality				.03	.02	.02
*R* ^2^	.42			.42		
Adjusted *R*^2^	.41			.42		
*F*	71.31**			66.63**		

*Note*. In Model 1, predictors are listed in descending order of beta coefficients.

We dummy-coded countries, choosing Italy as the reference category because it had the highest number of COVID-19 cases [40] and the strongest restrictions [41] at the time of assessment.

^a^ For the predictors, participants indicated their subjective level of change only (e.g., “*I see my partner slightly more than before*”), whereas for relationship satisfaction, they reported their respective levels before and after the onset of the pandemic, for which we calculated the residualized change score.

*p < .05.

**p < .01

^†^*p* < .10.

In a second step (Table 1, Model 2), we also included specific interactions to examine whether the association of changes in shared time (our strongest predictor) with changes in relationship satisfaction was moderated by enduring dispositions discussed in the introduction, namely, anxious attachment, avoidant attachment, and negative emotionality. Interaction terms were defined as a product of the respective *z*-transformed target variables.

**Fig 4:**
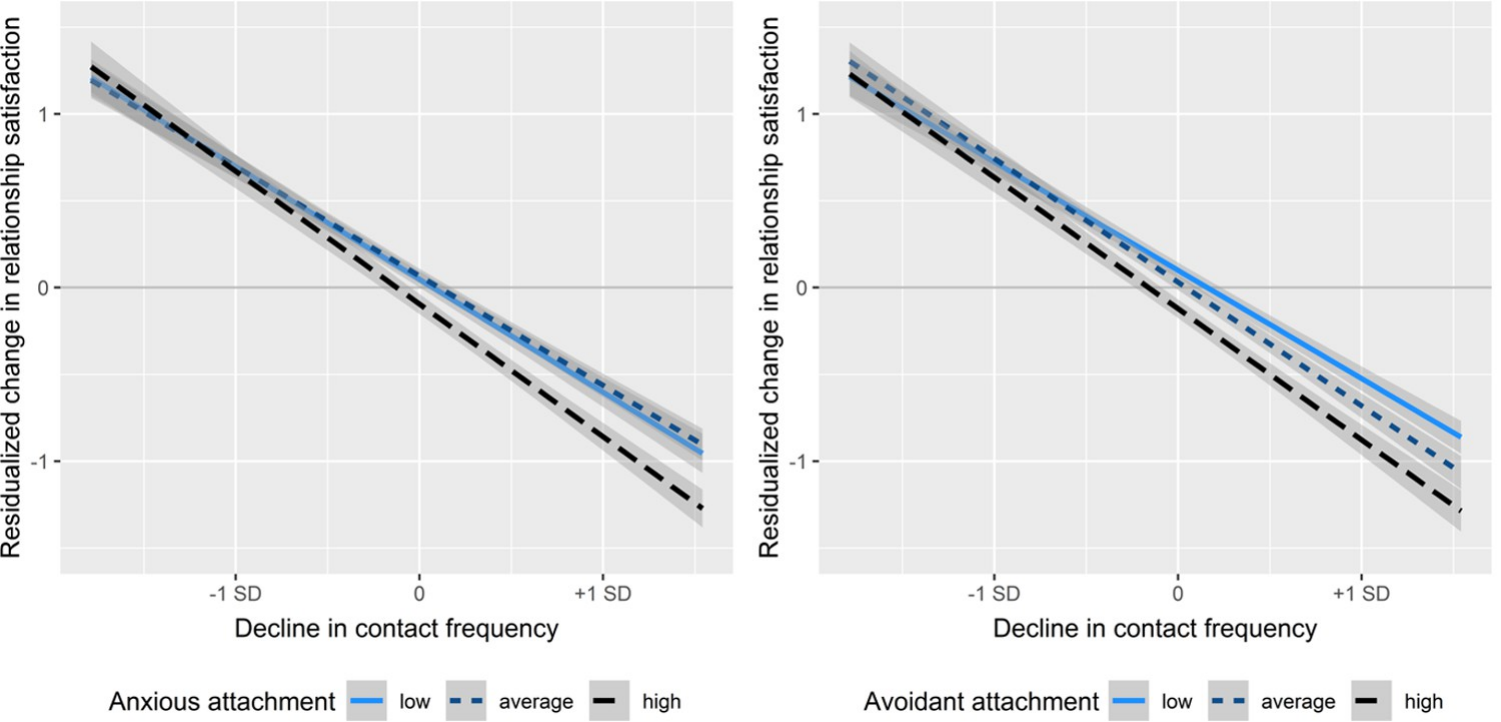
Moderation of the association between decrease in shared time and change in relationship satisfaction through avoidant and anxious attachment style. Decrease in shared time is the *z*-transformed composite contact variable. For purposes of visualization, we trichotomized the sample into high, medium, and low groups of insecure attachment.

Consistent with our expectations, higher levels of anxious attachment and avoidant attachment increased the negative effect of reduced contact on relationship satisfaction (see Table 1, Model 2), whereas negative emotionality showed neither main, nor moderating effects. Fig 4 shows this moderating effect of avoidant and anxious attachment on the association between change in shared time and perceived change in relationship satisfaction. The effect of less shared time is particularly strong for individuals with high levels of anxious and avoidant attachment.

#### Cohabiting and non-cohabiting individuals

As suggested by our earlier analyses, the situation of cohabiting and non-cohabiting individuals differed considerably in the early stages of the pandemic. As a consequence, we tested for significant interactions between cohabiting status and the predictor variables listed in Table 1, Model 1. A decrease in shared time was associated with a stronger perceived decrease in relationship satisfaction in non-cohabiting than in cohabiting individuals (β = -.12, *p* < .001). Conversely, an increase in arguing was associated with a stronger perceived decrease in relationship satisfaction for cohabiting than in non-cohabiting individuals (β = -.06, *p* = .004). An increase in time for oneself was associated with a perceived increase in relationship satisfaction in cohabiting individuals, but it was associated with a perceived decrease in relationship satisfaction in non-cohabiting individuals (β = .07, *p* = .001). S6 Table in S1 File, provides a summary of all tested interactions.

## Discussion

To our knowledge, this is the first study to use a large cross-national sample to examine relationship satisfaction at the beginning of the COVID-19 pandemic, compared with retrospectively assessed satisfaction before the pandemic, in both cohabiting and non-cohabiting individuals. Our results suggest a nuanced picture in which relationship satisfaction declined mostly for those who spent less time with their partners (especially non-cohabitants). Among those who spent as much or more time with their partner during the pandemic (e.g., cohabitants), the decline in relationship satisfaction was minimal. This result is consistent with the absence of notable changes in relationship satisfaction at the beginning of the pandemic recently found in a US study of cohabiting couples [4]. A German study, on the other hand, found a decline in relationship satisfaction among cohabiting married and unmarried couples, although relationship satisfaction was measured with only one item [5]. The mixed results seem to indicate that it is difficult to discern a clear trend in how cohabiting individuals responded to the pandemic. In line with the vulnerability-stress-adaptation model, we found insecure attachment, but not negative emotionality, to be related to a decline in relationship satisfaction. Similar results were obtained by Overall et al. [7], although in their study only the partner’s insecure attachment, and not the individual’s own attachment, predicted negative relationship functioning.

### Shared dyadic time

Our findings indicate that the perceived decrease in relationship satisfaction found among non-cohabiting individuals was largely driven by reduced time spent with the partner. Indeed, once shared time was controlled for, cohabiting status in and of itself was no longer a significant predictor. This result is consistent with previous findings showing that little to no face-to-face contact has a negative impact on relationship satisfaction in non-cohabiting partners [16]. The uncertainties about travel bans may have contributed to this decline and fueled doubts about the future of the relationship [42]. Another key factor that negatively affected relationship satisfaction among non-cohabiting individuals was a steep decline in sexual satisfaction. An obvious explanation for this trend is that travel bans and mobility restrictions subjected non-cohabitating individuals to prolonged periods of enforced sexual abstinence. Overall relationship satisfaction was also adversely affected in this group, but to a lesser extent, which indicates that general aspects of relationship quality (e.g., conversation, mutual support, humor) are more easily sustained without physical contact than is sexual activity.

Interestingly, a decrease in time spent with the partner was also associated with a decline in relationship satisfaction in cohabiting individuals, whereas an increase in shared time did not negatively affect their relationship satisfaction. Taken together, these results converge with previous work in suggesting that sharing activities with one’s partner is positively related to relationship satisfaction [43]. Furthermore, attachment theory assumes that adults seek closeness and support from close attachment figures as a reaction to stress [44]. In the case of an existential threat, closeness seems particularly critical in mitigating anxiety through mutual support and emotional comfort [24]. Travel restrictions and lack of physical contact may have prevented this mechanism from operating normally in non-cohabiting couples, providing an additional possible explanation for the larger decline in relationship satisfaction in this group.

Regarding the living situation, more time spent outside the home and more time for oneself (only among cohabiting individuals) were both positively related to relationship satisfaction. For people living together, alone time potentially provided a balance to the constant proximity.

### The vulnerability-stress-adaptation model

Among individual predispositions, we found that anxious and avoidant attachment was not only directly associated with a decline in relationship satisfaction, but that it was also indirectly associated through exacerbation of the negative effects of reduced shared dyadic time and activities. In an atmosphere of crisis and general uncertainty, the lack of opportunities for contact might have deprived the anxiously attached from much sought-after reassurance, while increasing the tendency of avoidant individuals to distance themselves in the face of mounting tensions. Contrary to our expectations, there was no relation between personality traits, including negative emotionality, and changes in relationship satisfaction. This might be due to the strong intercorrelations between measures of negative emotionality and insecure attachment, with the latter outperforming the Big Five dimensions in predicting relationship satisfaction [45].

### Mobility restrictions and relationship satisfaction

We were able to recruit an international sample through our survey, with most individuals coming from Italy, the United States, the United Kingdom, and the GSA region. A regression on the predictors of perceived change in relationship satisfaction showed that there was no effect of mobility restriction level or country; i.e., people from the different countries reported no significantly different changes in their relationship satisfaction. Of course, this result does not imply that ethnic, cultural, or sociodemographic factors were irrelevant. It is possible that cultural and sociodemographic factors may have been operating at a different level, such as at the level of culturally heterogeneous subgroups, which should be given closer attention in future work on the impact of the pandemic on romantic relationships.

### Limitations

In interpreting the findings, some limitations need to be considered. First, pre-pandemic data were assessed retrospectively, thus introducing possible bias into the comparison with in-pandemic data. This is a relatively common limitation of studies investigating the effects of unforeseen events. Similar approaches have been used in other studies on the perceived impact of the COVID-19 pandemic [25, 26], as well as in studies of other unforeseen events, such as the September 11, 2001, terror attacks [27]. The nature of recall bias in relationship research is not entirely clear. One study on recall bias in ratings of relationship satisfaction suggests that couples generally tend to rate past relationship satisfaction more negatively that it actually was [46], resulting in a trend for perceived satisfaction improvement rather than deterioration over time. Similarly, a study by Zygar-Hoffmann and Schönbrodt [28] showed that retrospective reports of annoyance toward the partner were overestimated, whereas the retrospective assessment of relationship satisfaction was quite accurate overall. If our results were affected by the recall bias as identified in previous research, they would have *under*estimated the degree of decline in relationship satisfaction. Moreover, a recall bias would presumably have affected cohabiting and non-cohabiting individuals in a similar manner. However, our findings showed pronounced differences that were consistent with expectations. Nonetheless, a real-time and a retrospective rating are clearly not the same thing, and this difference needs to be kept in mind when interpreting our results.

Second, because of time constraints and our aim at capturing the broadest possible range of influential predictors, we used brief scales with limited possibilities for in-depth analyses of individual constructs. Although some of the internal consistencies of the Big Five personality scales were quite low. this was not the case for neuroticism, which was the theoretically most relevant factor [35, 47].

Third, despite the large number of variables included, we cannot rule out the possibility that we may have omitted potentially important variables.

Fourth, although our samples were not palpably dissimilar to the respective general populations, they cannot be regarded as representative of these populations. Our sample had a slightly higher average level of education compared with the levels in the national populations, and so our results may particularly underestimate the impact of the pandemic on couples with a lower socioeconomic status, which was demonstrated by another study [6].

Fifth, the diversity of nations included in our research is a clear advantage of our study regarding the generalizability our findings. Still, one needs to keep in mind that there are also cultural differences within each nation and that an analysis of any such differences was not within the scope our research. Clearly, ethnic and cultural variations across nations are important and should be given adequate attention in future research. Finally, the current results relate to individuals rather than to couples, leaving the study of within-couple dynamics during the pandemic to future research. These limitations notwithstanding, the range of variables, countries, and types of relationships included in this research provides nuanced insights into how internal and external factors impinged on close relationships at the beginning of the COVID-19 pandemic.

All in all, what does this study tell us? First, our findings suggest that non-cohabiting individuals’ inability to meet regularly due to COVID-19-related mobility restrictions had a strong negative impact on their relationship. The man who crossed the Irish Sea from Scotland to the Isle of Man on a jet ski to visit his girlfriend, and was subsequently jailed for it, serves as a reminder of the degree of deprivation and urgency experienced by some couples [48]. To avert negative consequences for non-cohabiting couples in the future, governments should consider granting certain exemptions from travel restrictions to romantic partners, as had already been done by some governments [49, 50].

On the other hand, we found that the increased frequency of contact among cohabiting individuals was not necessarily associated with deteriorating relationship satisfaction. In these couples, what seemed to matter was to achieve a good balance between time together and time for oneself. Thus, cohabiting couples should not automatically expect negative consequences of being locked in together. Indeed, stay-at-home orders may have provided many couples a much-needed opportunity to spend more time together, forging a renewed sense of togetherness. Because negative expectations can turn into self-fulfilling prophecies, conveying this message to a broader audience is important.

Third, previous research on relationships and stress has identified several factors that affect relationship satisfaction. However, whether these factors would also have similar protective or harmful effects during an unanticipated major global crisis such as the current pandemic remained unclear. Some authors have argued that this might be the case and that models of relationship functioning such as the vulnerability-stress-adaptation model ought to also provide valid predictions for the exceptional case of the pandemic [3]. Some of our findings, notably those about the role of attachment, offer initial empirical evidence in support of this assumption, thereby filling an important gap in research on relationship functioning. Whether this initial evidence will generalize to longer term effects of the pandemic on relationship satisfaction is a matter for future research. Findings related to later phases of the pandemic will be crucial for obtaining a more complete understanding of the effects of the pandemic on close relationships and for developing appropriate preventive policies.

## Supporting information

S1 File (DOCX)
