# Supplementary material for: Relationship satisfaction in the early stages of the COVID-19 pandemic: A cross-national examination of situational, dispositional, and relationship factors
Source: PLoS One. 2022 Mar 3;17(3):e0264511. doi: 10.1371/journal.pone.0264511 (PMC8893701; doi:10.1371/journal.pone.0264511)
Supplement: S1 File — (DOCX) [file pone.0264511.s001.docx]

Relationship Satisfaction in the Early Stages of the COVID-19 Pandemic:

A Cross-National Examination of Situational, Dispositional, and Relationship Factors

**Supplemental Online Material**

Contents

[Comparison of Sample to Population 2](#_Toc93048714)

[Unpaid and Paid (Clickworker) Sample (United States and United Kingdom) 3](#_Toc93048715)

[Pandemic-Related Statistics 4](#_Toc93048716)

[Changes in Relationship Satisfaction (Relationship Assessment Scale Items) and Sexual Satisfaction 5](#_Toc93048717)

[Factors Associated With Perceived Change in Relationship Satisfaction Among Cohabiting and Non-Cohabiting individuals 6](#_Toc93048718)

[References 8](#_Toc93048719)

# Comparison of Sample to Population

**Table S1.** Comparison Between Population and Sample Characteristics

| Variable | Specification | Country^a^ | Our sample^b^ | Population | Source |
| --- | --- | --- | --- | --- | --- |
| Gender | Women | Italy  GSA region  United States  United Kingdom | 74.5%  87.5%  66.1%  36.1% | 51.3%  50.6%  50.5%  50.6% | The World Bank (2019) |
| Education | Bachelor’s, master’s, or doctoral degree | Italy  GSA region  United States  United Kingdom | 36%  56%  73%  68% | 28%  42%  40%  44% | OECD (2020) |
| Sexual orientation | Heterosexual | Italy  GSA region  United States  United Kingdom | 82%  89%  83%  85% | 87%  87%  88%  91% | Rahman et al. (2020) |
|  | Bisexual | Italy  GSA region  United States  United Kingdom | 13%  6%  11%  8% | 8%  8%  7%  5% |  |
|  | Homosexual | Italy  GSA region  United States  United Kingdom | 2%  5%  5%  5% | 5%  4%  5%  4% |  |
| Cohabiting status | Non-cohabiting | Italy  GSA region  United States  United Kingdom | 67%  37.4%  23.1%  34.1% | Approximately 10% in Western Europe and North America | Duncan and Phillips (2011), Reuschke (2010) |
|  |  |  | **Median** | **Median** | **Source** |
| Age | Median Age | Italy  GSA region  United States  United Kingdom | 24  26  36  30 | 47.3  44.1  38.3  40.5 | United Nations, Department of Economic and Social Affairs (2019) |
|  |  |  |  |  |  |
|  |  |  | **Mean (SD)** | **Mean (SD)** | **Source** |
| Relationship satisfaction^b^ | Relationship Assessment Scale (Hendrick, 1988) | Italy  GSA region  United States  United Kingdom | 4.26 (0.70)  4.27 (0.71)  4.07 (0.86)  4.10 (0.80) | -  4.29 (0.59)  4.16 (0.92)  4.29 (0.71) | -  Dinkel and Balck (2005)  Hendrick (1988)  Cramer (2001) |

^a^*n*_Italy_ = 1,094; *n*_German-speaking countries_ = 775; *n*_US_ = 776; *n*_UK_= 302. ^b^For comparison with the norms, retrospectively assessed relationship satisfaction (before the pandemic) was used. Hendrick (1988) reported means and standard deviations for the single items; we calculated means from these.

# Unpaid and Paid (Clickworker) Sample (United States and United Kingdom)

**Table S2.** Comparison of the Clickworker Samples and the Respective National Samples

| Variable | Range | Country | Unpaid | Clickworker | *df* | *p* | *t* |
| --- | --- | --- | --- | --- | --- | --- | --- |
| Age | 18 to 79 | US  UK | 39.47 (13.20)  32.30 (10.89 | 35.21 (9.32)  35.78 (10.79) | 642.65  299 | <.001  .004 | 5.15  -2.87 |
| Education^a^ | 0 to 4 | US  UK | 3.18 (0.83)  2.73 (0.98) | 2.57 (0.89)  2.69 (1.00) | 774  299 | <.001  .740 | 9.19  0.33 |
| Relationship duration | - 1. to 5.6 | US  UK | 9.98 (10.79)  5.91 (6.92) | 7.80 (8.17)  7.97 (9.48) | 605.29  274.56 | .002  .033 | 3.10  -2.15 |
| Decline in contact frequency | -1.8 to 1.5 | US  UK | -0.26 (0.67)  -0.09 (0.86) | -0.30 (0.71)  0.18 (0.78) | 774  299 | .372  .314 | 0.89  1.01 |
| Residualized change in relationship satisfaction | -5 to 4 | US  UK | 0.18 (1.03)  0.00 (1.09) | 0.16 (0.86)  -0.08 (0.89) | 774  299 | .883  .509 | 0.15  0.66 |

*Note.* Standard deviations are given in parentheses.

^a^0 = less than high school; 1 = high school/A-levels; 2 = some college, no degree; 3 = bachelor’s degree; 4 = master’s and/or PhD degree.

# Pandemic-Related Statistics

**Table S3a.** Change in Physical Contact Frequency

| Answer options | Non-cohabiting | Cohabiting |
| --- | --- | --- |
| Less than half of the time | 71.2% [1,010] | 1.3% [23] |
| About half of the time | 3.9% [56] | 0.6% [11] |
| Slightly less than before | 5.5% [78] | 1.9% [35] |
| About the same as before | 6.5% [92] | 11.2% [205] |
| Slightly more than before | 5.4% [77] | 21.2% [386] |
| About twice as much | 3.2% [46] | 23.6% [430] |
| More than twice as much | 4.2% [59] | 40.3% [735] |

*Note. n* is given in square brackets.

**Table S3b.** Occupational Situation

| Participants characteristics | Overall | Men | Women |
| --- | --- | --- | --- |
| Were occupied before the pandemic | 67.1% [2,176] | 77.1% [661] | 63.7% [1,515] |
| Were occupied during the pandemic | 60.5% [1,962] | 68.8% [594] | 57.5% [1,368] |
| The occupational situation changed during the pandemic (only participants that worked before the pandemic, *n* = 2,176) | 76.9% [1,673] | 74.0% [489] | 78.2% [1,184] |
| Type of change (only participants whose work situation has changed, *n* = 1,673) |  |  |  |
| Work more from home | 49.1% [822] | 50.9% [249] | 48.4% [573] |
| Reduced working hours | 27.8% [465] | 29.7% [145] | 27% [320] |
| Increased working hours | 12.1% [203] | 11.7% [57] | 12.3% [146] |
| Leave of absence | 7.4% [124] | 8.8% [43] | 6.8% [81] |
| The workplace closed | 16.6% [277] | 13.9% [68] | 17.7% [209] |
| Fired because of the pandemic | 6.3% [105] | 6.3% [31] | 6.3% [74] |
| The contract has expired | 1.1% [19] | 1.2% [6] | 1.1% [13] |
| Has quit work | 1.3% [21] | 1.4% [7] | 1.2% [14] |
| Found a new job | 2.4% [40] | 1.8% [9] | 2.6% [31] |
| Other | 18.1% [303] | 18.6% [91] | 17.9% [212] |

*Note. n* is given in square brackets.

**Table S3c.** Time (in Hours) Per Week Spent Outside

| Outdoor time | Overall | | Men | | Women | |
| --- | --- | --- | --- | --- | --- | --- |
|  | *M* | *SD* | *M* | *SD* | *M* | *SD* |
| At work | 6.15 | 13.39 | 8.53 | 15.92 | 5.28 | 12.25 |
| For leisure activities | 3.85 | 6.17 | 4.69 | 7.27 | 3.54 | 5.69 |
| For other activities | 2.24 | 3.78 | 3.06 | 4.18 | 1.94 | 3.58 |
| Total | 12.23 | 16.82 | 16.27 | 19.42 | 10.76 | 15.51 |

**Table S3d.** Worries Regarding Different Aspects of COVID-19

| Worries regarding | Overall | | Men | | Women | |
| --- | --- | --- | --- | --- | --- | --- |
|  | *M* | *SD* | *M* | *SD* | *M* | *SD* |
| Spreading of the virus | 6.15 | 2.48 | 5.66 | 2.61 | 6.33 | 2.41 |
| Health of others | 7.01 | 2.52 | 6.65 | 2.62 | 7.14 | 2.47 |
| One’s own health | 4.35 | 2.71 | 4.05 | 2.65 | 4.45 | 2.72 |
| Work-related worries | 3.97 | 3.07 | 4.15 | 3.08 | 3.91 | 2.07 |
| Financial worries | 4.34 | 3.03 | 4.33 | 3.05 | 4.34 | 3.02 |
| Social worries | 3.20 | 2.36 | 3.33 | 2.41 | 3.15 | 2.34 |

*Note*. Respondents indicated answers on a scale that ranged from 1 (*not at all*) to 10 (*very worried*).

**Table S3e.** Self-Reported Restriction Levels

| Country | No restrictions | Some restrictions | Moderate restrictions | Severe restrictions |
| --- | --- | --- | --- | --- |
| Italy  United Kingdom | -  0.7% | 0.3%  2% | 2.2%  46.8% | 97.5%  71.5% |
| GSA region  United States | 0.1%  - | 2.3%  2.6% | 48.3%  46.8% | 49.3%  50.6% |

# Changes in Relationship Satisfaction (Relationship Assessment Scale Items) and Sexual Satisfaction

**Table S4.** Mixed Repeated Measures ANOVA: Changes in Relationship Satisfaction and Sexual Satisfaction Among Cohabiting and Non-Cohabiting Individuals.

| Effects | *df* | *F* | Sig. | η^2^ |
| --- | --- | --- | --- | --- |
| Between-subject effects  Cohabiting | 1, 3241 | 52.46 | < .001 | .02 |
| Within-subject effects  Relationship satisfaction time  Relationship satisfaction time × Cohabiting  Sexual satisfaction time  Sexual satisfaction time × cohabiting  Relationship satisfaction time × Sexual satisfaction time  Relationship satisfaction time × Sexual satisfaction time  × Cohabiting | 1, 3241  1, 3241  1, 3241  1, 3241  1, 3241  1, 3241 | 1595.90  25.59  1521.69  1051.92  1070.96  734.88 | < .001  < .001  < .001  < .001  < .001  < .001 | .33  .01  .32  .25  .29  .19 |
| Note. n cohabiting = 1,825; n non-cohabiting = 1,418. | | | | |

**Table S5.** Changes in Single RAS Items and the Item on Sexual Satisfaction Among Cohabiting and Non-Cohabiting Individuals

| Item | Group | T0 | T1 | *df* | *t* | *p* | *d* |
| --- | --- | --- | --- | --- | --- | --- | --- |
| How well does your partner meet your needs? | Cohabiting  Non-cohabiting | 3.98  4.15 | 3.94  3.37 | 1824  1417 | 2.12  23.23 | .034  < .001 | 0.10  1.23 |
| In general, how satisfied are you with your relationship? | Cohabiting  Non-cohabiting | 4.18  4.22 | 4.07  3.68 | 1824  1417 | 5.65  18.76 | < .001  < .001 | 0.27  1.00 |
| How good is your relationship compared to most? | Cohabiting  Non-cohabiting | 4.19  4.17 | 4.13  3.82 | 1824  1417 | 3.67  13.30 | < .001  < .001 | 0.17  0.71 |
| How often do you wish you had not gotten in this relationship? (reversed) | Cohabiting  Non-cohabiting | 4.43  4.57 | 4.46  4.52 | 1824  1417 | -2.09  3.11 | .037  .002 | -0.10  0.17 |
| To what extent has your relationship met your original expectations? | Cohabiting  Non-cohabiting | 4.00  4.07 | 3.98  3.62 | 1824  1417 | 0.92  15.73 | .359  < .001 | -  0.84 |
| How much do you love your partner? | Cohabiting  Non-cohabiting | 4.60  4.49 | 4.56  4.45 | 1824  1417 | 3.94  3.01 | < .001  .003 | 0.19  0.16 |
| How many problems are there in your relationship? (reversed) | Cohabiting  Non-cohabiting | 3.95  4.02 | 3.94  3.79 | 1824  1417 | 0.52  9.02 | .602  < .001 | -  0.48 |
| How sexually satisfied are you with your partner? | Cohabiting  Non-cohabiting | 3.57  4.03 | 3.41  2.34 | 1824  1417 | 7.46  38.42 | < .001  < .001 | 0.35  2.04 |

Note. T0 = before the pandemic; T1 = during the pandemic; n cohabiting = 1,825; n non-cohabiting = 1,418. RAS = Relationship Assessment Scale.

# Factors Associated With Perceived Change in Relationship Satisfaction Among Cohabiting and Non-Cohabiting individuals

**Table S6.** Factors Associated With Residualized Change in Relationship Satisfaction and Interactions with Cohabiting, Listed in the Same Order as in Table 1 in the Manuscript.

| Variable | *B* | *SE B* | β |
| --- | --- | --- | --- |
| Decrease in shared time | -.84 | .03 | -.68** |
| Increase in arguing | -.10 | .01 | -.20** |
| Pandemic-related worries | -.02 | .01 | -.03 |
| Avoidant attachment | -.06 | .02 | -.09** |
| Increase in time for oneself | < .01 | .01 | .01 |
| Feeling of privacy at home | .05 | .02 | .06** |
| Relationship duration | .01 | < .01 | .05* |
| Anxious attachment | -.03 | .01 | -.05* |
| Decrease in working hours (self) | -.11 | .05 | -.05* |
| Hours per week spent outside home | < .01 | < .01 | .02 |
| Living in the GSA region | .04 | .06 | .02 |
| Living in the US | .08 | .08 | .03 |
| Loss of employment (partner) | -.10 | .07 | -.03 |
| Living in the UK | -.08 | .06 | -.02 |
| Decrease in working hours (partner) | -.11 | .09 | -.03 |
| Cohabiting | .13 | .16 | .06 |
| Living in other countries | .06 | .06 | .02 |
| Agreeableness | -.05 | .03 | -.03 |
| Loss of employment (self) | -.10 | .06 | -.03 |
| Size of the living area | -.04 | .03 | -.03 |
| Home size | < .01 | < .01 | -.01 |
| Education | -.01 | .02 | -.02 |
| Restriction level of the country | .05 | .05 | .03 |
| Extraversion | -.04 | .03 | -.03 |
| Gender | .02 | .05 | .01 |
| Living with children | .03 | .09 | .01 |
| Duration of pandemic-related restrictions | < .01 | < .01 | .02 |
| Conscientiousness | -.02 | .03 | -.01 |
| Access to a private outdoor space | .04 | .07 | .02 |
| Openness | -.03 | .03 | -.02 |
| Negative emotionality | -.03 | .03 | -.03 |
| Age | < .01 | < .01 | .01 |
| Decrease contact frequency × Cohabiting | -.25 | .05 | -.12** |
| Increase in arguing × Cohabiting | -.08 | .03 | -.06** |
| Pandemic-related worries × Cohabiting | -.05 | .03 | -.04 |
| Avoidant attachment × Cohabiting | .05 | .03 | .04 |
| Increase in time for oneself × Cohabiting | .11 | .03 | .07** |
| Feeling of privacy at home × Cohabiting | -.02 | .03 | -.01 |
| Relationship duration × Cohabiting | -.04 | .03 | -.03 |
| Anxious attachment × Cohabiting | .01 | .03 | .01 |
| Decrease in working hours (self) × Cohabiting | .02 | .03 | .01 |
| Hours per week spent outside home × Cohabiting | .01 | .03 | .01 |
| Living in the GSA region × Cohabiting | .03 | .08 | .01 |
| Loss of employment (partner) × Cohabiting | .01 | .03 | .01 |
| Living in the UK × Cohabiting | -.01 | .09 | < .01 |
| Decrease in working hours (partner) × Cohabiting | .01 | .03 | .01 |
| Agreeableness × Cohabiting | .03 | .03 | .02 |
| Loss of employment (self) × Cohabiting | .03 | .03 | .02 |
| Size of the living area × Cohabiting | .02 | .03 | .01 |
| Home size × Cohabiting | -.01 | .03 | -.01 |
| Education × Cohabiting | .01 | .03 | .01 |
| Restriction level × Cohabiting | -.02 | .03 | -.01 |
| Extraversion × Cohabiting | .04 | .03 | .03 |
| Gender × Cohabiting | -.04 | .07 | -.03 |
| Living with children × Cohabiting | -.01 | .10 | < .01 |
| Conscientiousness × Cohabiting | < .01 | .03 | < .01 |
| Access to a private outdoor space × Cohabiting | -.04 | .08 | -.02 |
| Openness × Cohabiting | .03 | .03 | .03 |
| Negative emotionality × Cohabiting | .06 | .03 | .04 |
| Age × Cohabiting | < .01 | < .01 | -.05 |

*Note*. DV = residualized change in relationship satisfaction;

Constant = 1.24; adjusted *R*^2^ = .417; *F* = 39.66, *p* < .001;

**p* < .05. ***p* < .01.

References

Duncan, S., & Phillips, M. (2011). People who live apart together (lats): New family form or just a stage? *International Review of Sociology*, *21*(3), 513–532. https://doi.org/10.1080/03906701.2011.625660

OECD. (2020). *Population with tertiary education* [indicator]. https://data.oecd.org/eduatt/population-with-tertiary-education.htm#indicator-chart

Rahman, Q., Xu, Y., Lippa, R. A., & Vasey, P. L. (2020). Prevalence of sexual orientation across 28 nations and its association with gender equality, economic development, and individualism. *Archives of Sexual Behavior*, *49*(2), 595–606. https://doi.org/10.1007/s10508-019-01590-0

Reuschke, D. (2010). Living apart together over long distances—time-space patterns and consequences of a late-modern living arrangement. *ERDKUNDE*, *64*(3), 215–226. https://doi.org/10.3112/erdkunde.2010.03.01

United Nations, Department of Economic and Social Affairs. (2019). *World population prospects 2019: volume ii: Demographic profiles (st/esa/ser.A/427)*. https://population.un.org/wpp/Publications/Files/WPP2019_Volume-II-Demographic-Profiles.pdf

The World Bank. (2019). *Population, female (% of total population): world bank staff estimates based on age/sex distributions of United Nations population division's world population prospects: 2019 revision.* https://data.worldbank.org/indicator/SP.POP.TOTL.FE.ZS?end=2020&start=1960&view=chart
